# Supplementary material for: RUFY3 regulates endolysosomes perinuclear positioning, antigen presentation and migration in activated phagocytes
Source: Nat Commun. 2023 Jul 18;14:4290. doi: 10.1038/s41467-023-40062-x (PMC10354229; doi:10.1038/s41467-023-40062-x)
Supplement: Supplementary file 1 — Supplementary Information [file 41467_2023_40062_MOESM1_ESM.pdf]

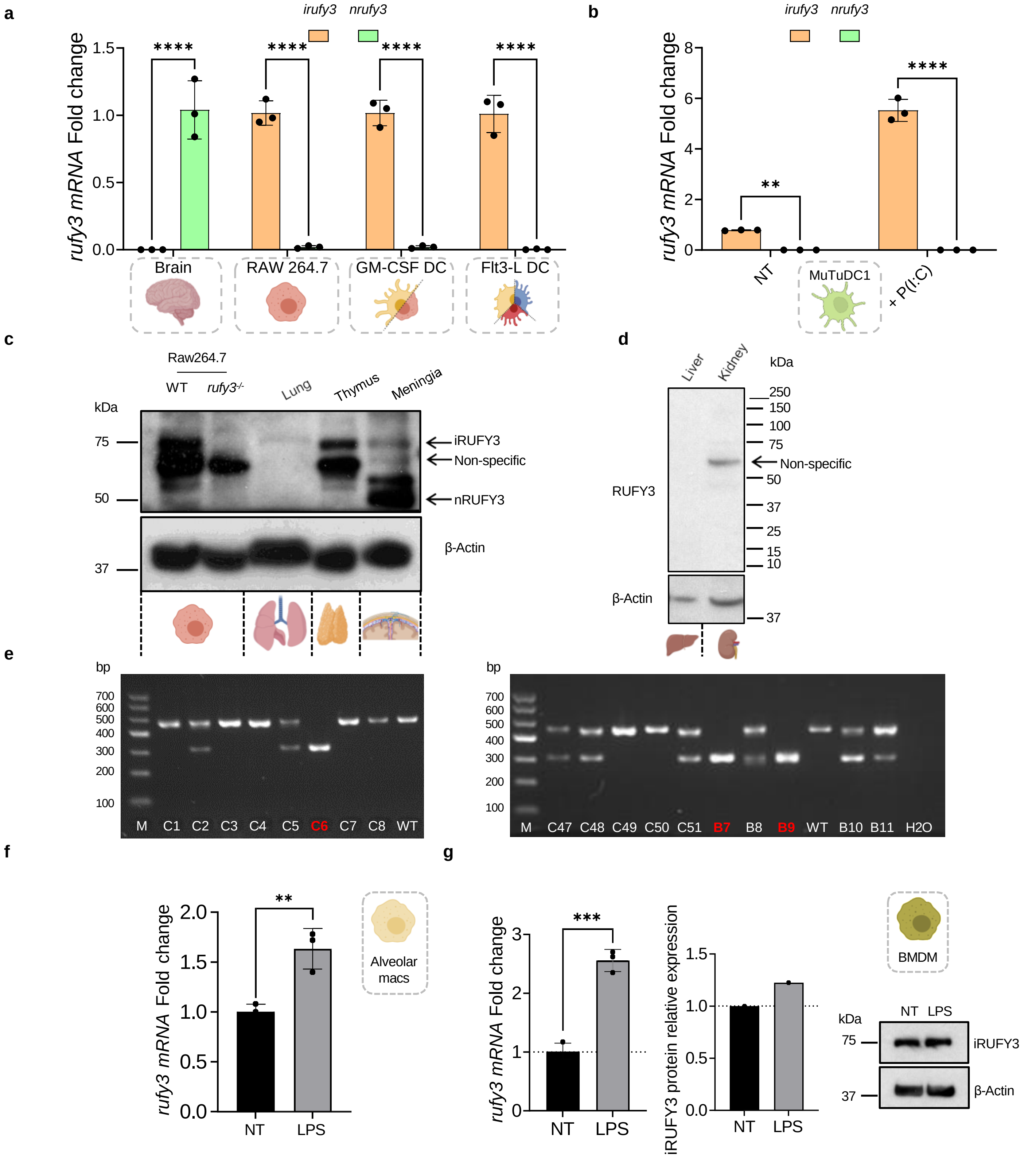

Supplementary Figure 1: The FYVE domain-bearing RUFY3 isoform is mostly found in immune cells and regulated by microbial or Interferon activation.

a-b) Comparative expression levels of the different *rufy3* mRNA isoforms in brain, Raw264.7 (RAW), bone marrow-derived DCs (GM-CSF and FLT3-L) at steady state **(a)** and in MuTuDC1 cell line with or without TLR3 stimulation with P(I:C) (8h, 5µg/mL) **(b)**. Data are presented as mean values +/- SD. **c-d)** Immunoblot detection of RUFY3 in Raw264.7 cells (RAW), RAW *rufy3*$^{-/-}$, total lung, thymus or meningia extracts (**c**) and in liver, kidney extract (**d**). Blots are representative of two independent experiments. **e**) Agarose gel electrophoresis analysis of PCR amplification products of the *rufy3* gene targeted by CRISPR/cas 9 in RAW cells. Clones bearing deletion in the 450bp wt *rufy3* sequence (C6, B7 and B9 in red) were selected and isolated for functional analysis as RAW *rufy3*$^{-/-}$. M corresponds to DNA Marker I. Screening is representative of two independent experiments. **f)** *rufy3* mRNA expression in alveolar macrophages with or without LPS stimulation (100ng/mL). Data are presented as mean values +/- SD. **g)** *rufy3* mRNA expression at mRNA level (left) and protein level (right) in Bone Marrow Derived Macrophages (BMDM) with or without 16h LPS stimulation (100ng/mL). Statistical significance was established using one and two-way ANOVA test (* p<0,05; **** p<0,0001). n=3 independent experiments. Data are presented as mean values +/- SD.

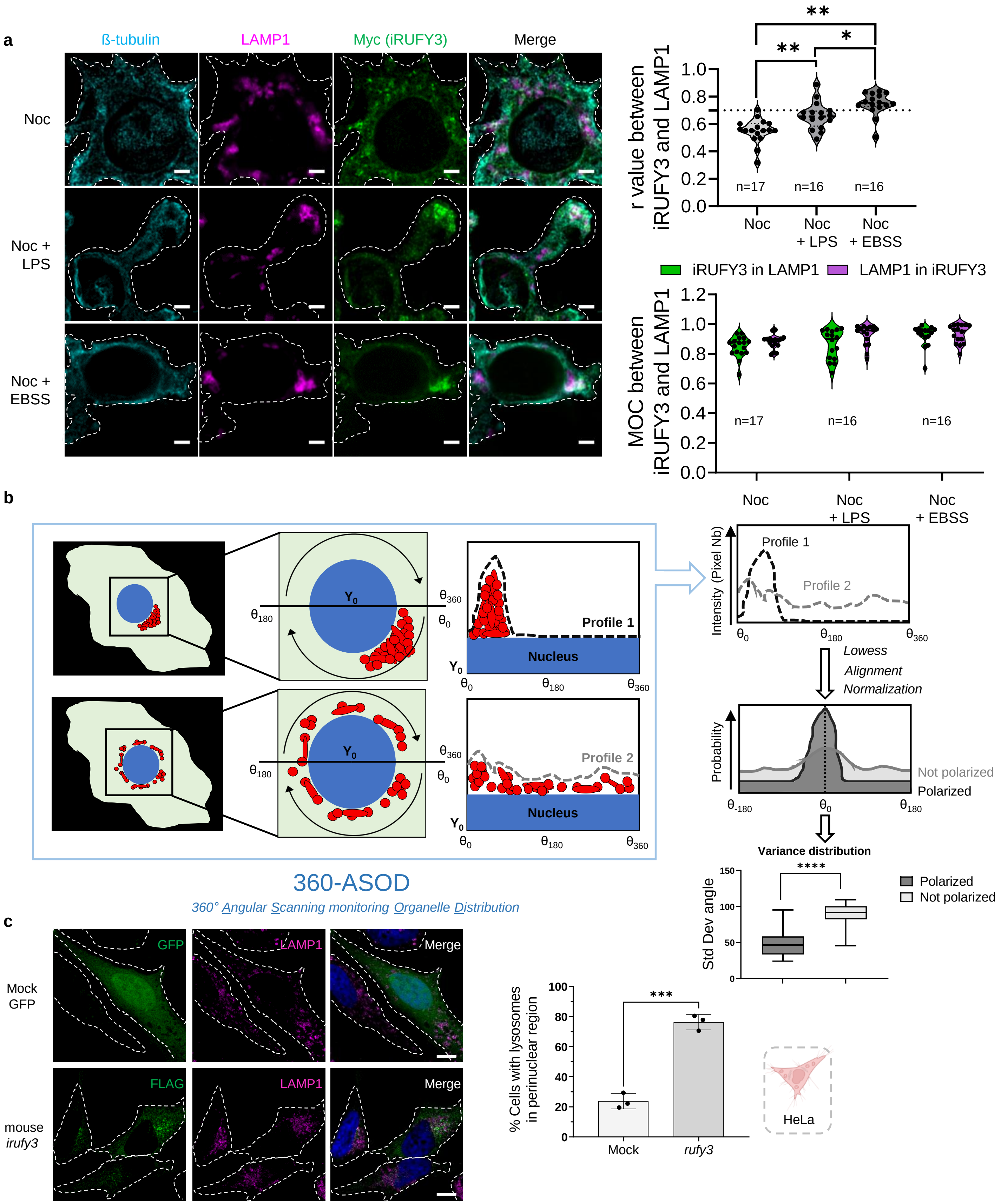

Supplementary Figure 2. Rufy3 promotes LAMP1+ vesicles clustering in the pericentriolar region

a) RAW-iRUFY3 cells were treated with Nocodazole (Noc, 10µM, 6h) prior detection of myc-RUFY3 and LAMP1+ EL by AICM. Dotted lines indicate cell boundaries. Pearson's co-localization and Mander's Overlay Coeficients (MOC) were calculated using Image J. A clear co-localization score is above 0.7 for Pearson's. Each dot corresponds to a cell**.** Statistical significance was established using unpaired t-test. * p<0,05, ** p<0,01. **b)** Graphical abstract of clustering indexes and 360-ASOD calculation methods to measure organelles dispersion. The boxplot illustrates examples of data obtained with 360°-ASOD. Data represent medians, interquartile ranges and spikes to upper and lower adjacent values. **c)** Detection of myc-RUFY3 and LAMP1+ EL by AICM in HeLa cells transiently overexpressing iRUFY3 and quantification of LAMP1+EL perinuclear clustering. Scale bar means 10 µm. Data obtained from n=3 independent experiments with 100 cells for each experiment. Statistical significance was established using unpaired Welch's t-test (*** p=0,0002). Data are presented as mean values +/- SD.

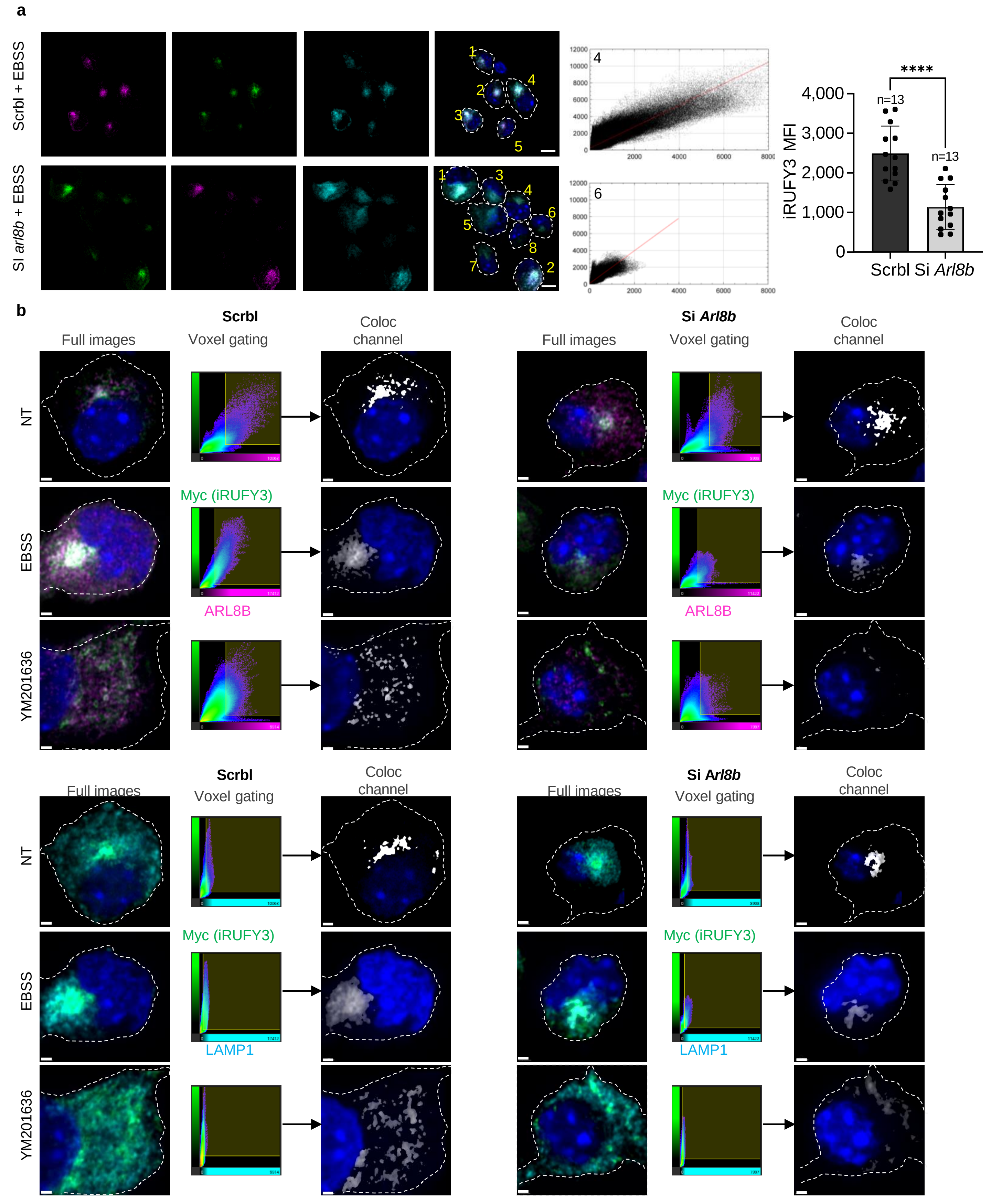

Supplementary Figure 3. iRUFY3 and ARL8b expression levels are correlated.

a) iRUFY3, ARL8B and LAMP1 imaging by AICM on RAW-iRUFY3 cells transfected with scramble RNA or Si RNA against Arl8b. Dotted lines indicate cell boundaries. Scale bar is 5µm. Cytofluorograms representing the intensity for each iRUFY3 and ARL8B voxels on two representative staining were extracted from ImageJ. Mean Fluorescent of Intensity (MFI) for iRUFY3 staining was extracted for each cell. Each dot corresponds to one cell (n=13). Statistical relevance was quantified with t-test (* p<0,05). Data are presented as mean values +/- SD. **b)** Voxel gating and co-localization channel isolation showing overlapping areas between iRUFY3 and ARL8B (top) or LAMP1 (bottom) in control (Scrbl) and Arl8b-silenced cells at steady state, under nutrient deprivation (EBSS, 6h) or with PIKfyve inhibitor (YM201636, 5µM for 45min). Scale bar is 1µm. Images are representative of two independent experiments with 100 cells for each experiment.

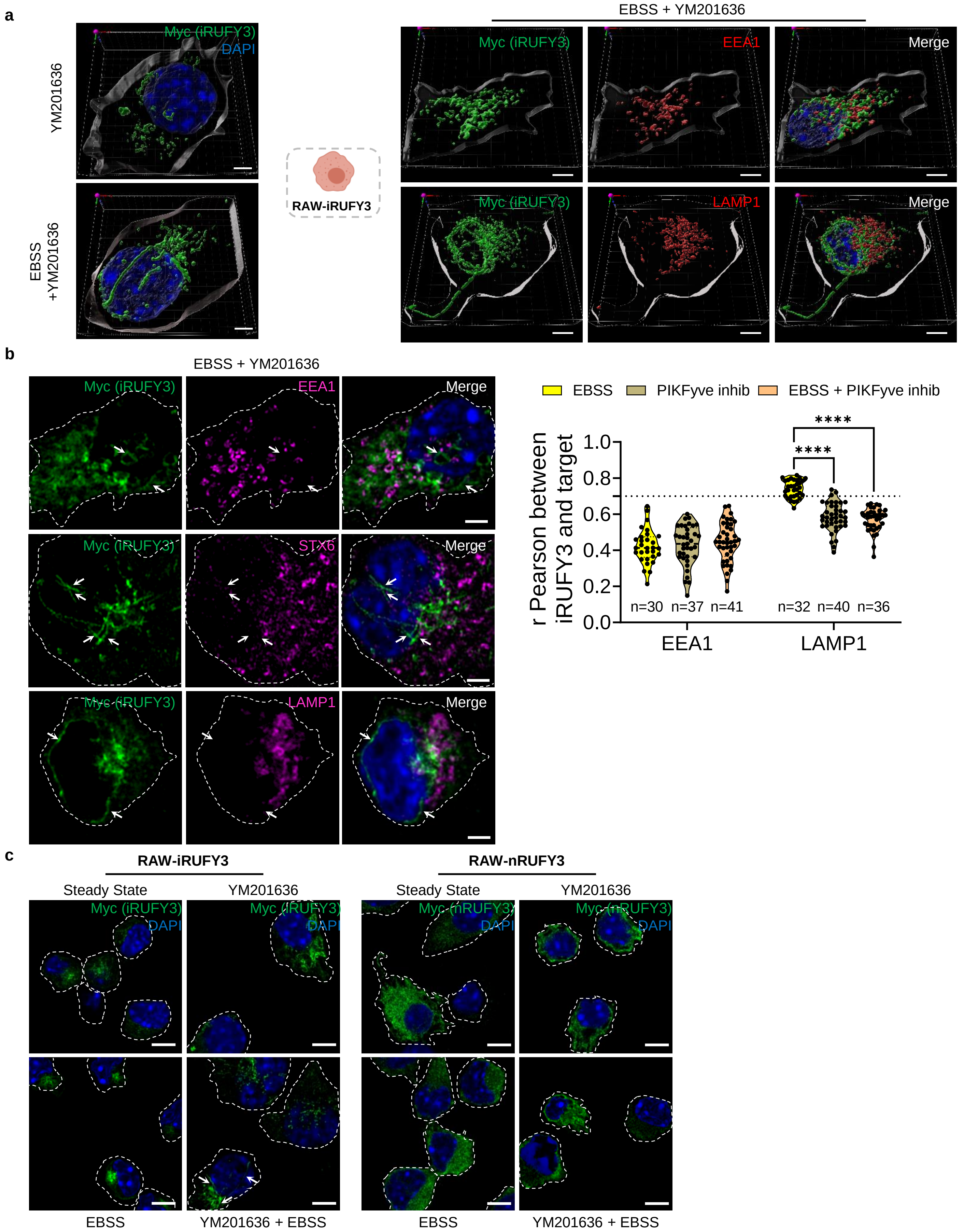

Supplementary Figure 4. iRUFY3 decorates large membrane tubules upon PtdIns(3)P accumulation.

a) 3D display of iRUFY3+ tubules after PIKfyve inhibitor treatment (left) and EBSS starvation (right). Scale bar 2µm. **b)** iRUFY3+ tubules intracellular distribution by AICM compared to endocytic markers EEA1, STX6 and LAMP1 upon YM201636 treatment (5µM for 45 min) and 6h of starvation (EBSS). Scale bar 3µm. Each dot represents the mean off all Z-stack from one region of interest. Statistical relevance was established using two-way ANOVA with Tukey's multiple comparisons test (* $p<0,05$; ** $p<0,01$). **c)** Detection of myc-tagged RUFY3 by AICM in RAW-iRUFY3 (left) and RAW-nRUFY3 (right) at steady state and YM201636 treatment (5µM for 45 min). Cells were also starved for 6h in EBSS in same conditions (bottom panels). Scale bar 5µm. Images are representative of three independent experiments with 100 cells for each experiment.

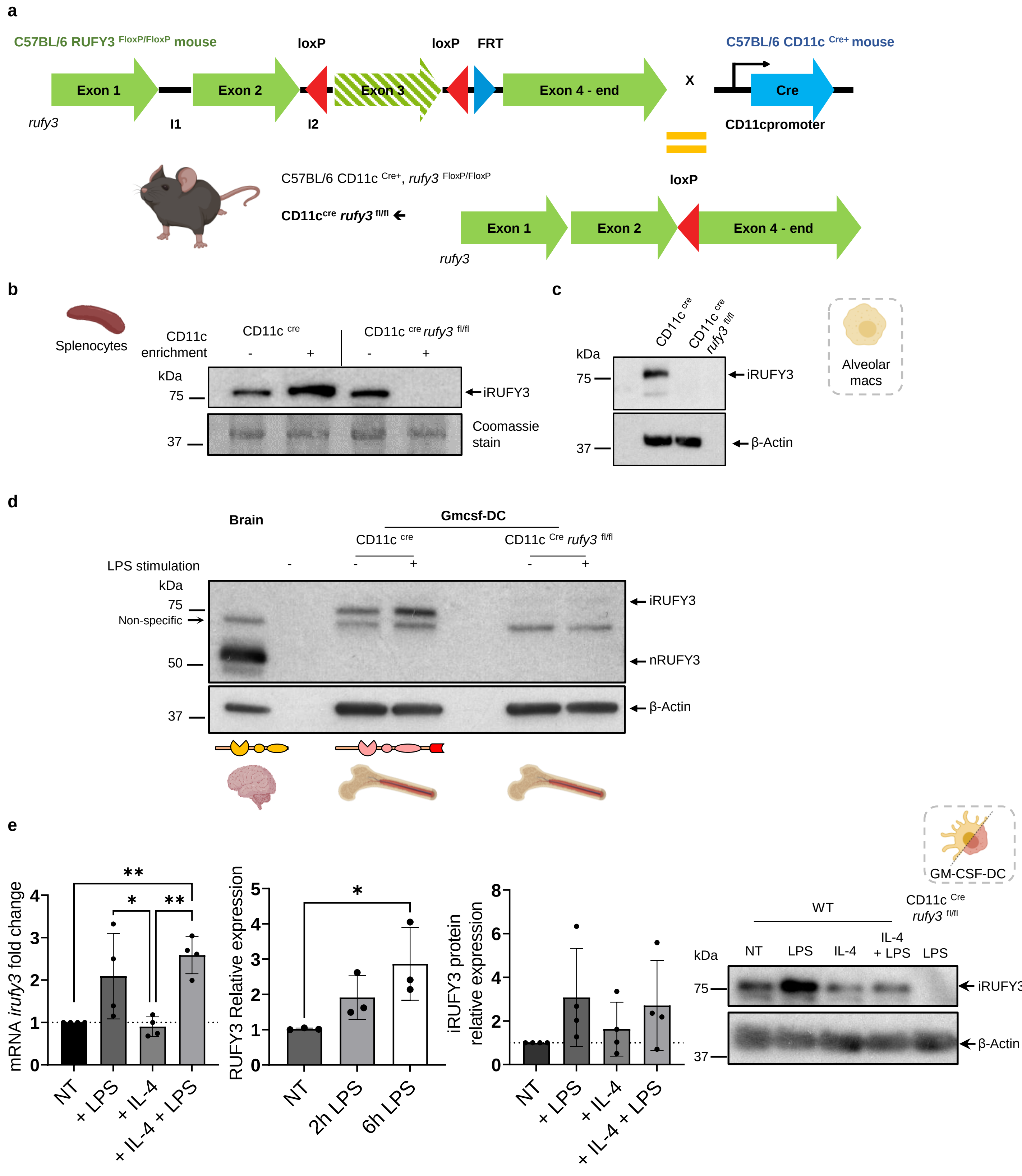

Supplementary Figure 5. Generation of the CD11c^{cre} rufy3 $^{fl/fl}$ mouse model.

a) The *rufy3* gene is constituted by 18 exons. The *rufy3* $^{loxp/loxp}$ mice was generated using a combination of Cre/loxP and FRT/FLP systems. *rufy3* $^{loxp/loxp}$ was developed by introducing a neomycin resistance cassette flanked by two loxP sequences in the second *rufy3* exon. Mice were bred to Itgax-Cre+ mice[47] to generate CD11c^{cre}*rufy3* $^{fl/fl}$ in which the RUFY3 protein is not expressed in cells co-expressing the integrin CD11C. **b-d)** loxP excision and iRUFY3 extinction was confirmed by immunoblot for cell lysates obtained from spleen (**b**), alveolar macrophages (AM) (**c**) and GM-CSF bmDCs (**d**). Blots are reprensetative of three independent experiments. **e)** Expression of *rufy3* mRNA in WT and CD11c^{cre}*rufy3* $^{fl/fl}$ GM-CSF bmDCs stimulated with LPS and/or differentiated with IL-4 was confirmed by RT-qPCR, while iRUFY3 protein expression loss was demonstrated by immunoblot. Data are of n=3 independent experiments and presented as mean values +/- SD. Statistical relevance was established using one-way ANOVA with Tukey's multiple comparisons test (* p<0,05; **p<0,01).

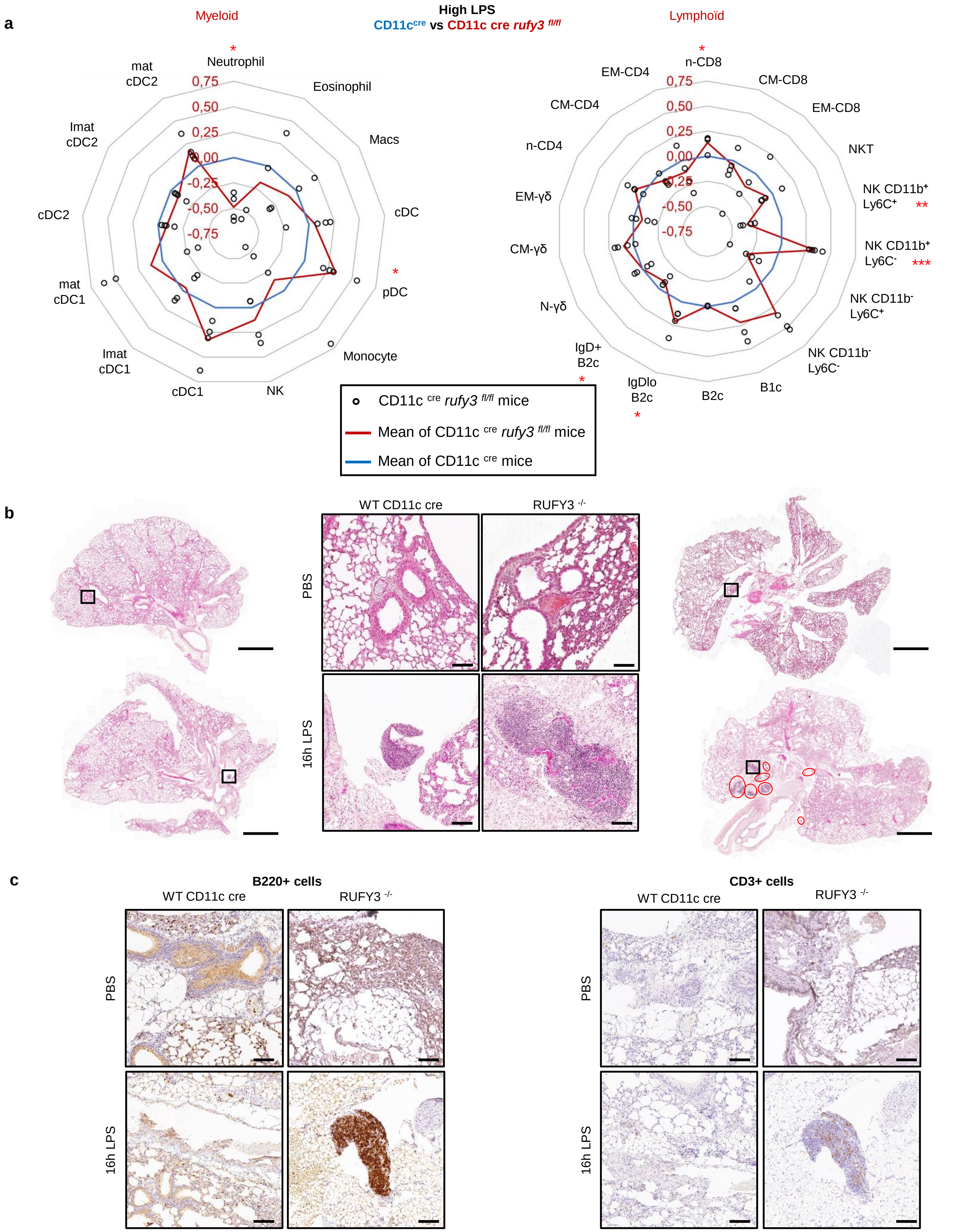

Supplementary Figure 6 Rufy3 deletion induce inflammation and B cell infiltration in lung

a) Immunophenotyping of myeloid (left) and lymphoid (right) splenocytes populations from WT and CD11c^{cre} *rufy3*$^{fl/fl}$ mice by flow cytometry with high dose LPS (10µg) injection. For experiment, 3 CD11c ^{cre} mice and 4 CD11c^{cre} *rufy3* $^{fl/fl}$ were used. **b)** Hematoxylin and eosin staining from lung sections reveals immune cells infiltration and clustering in CD11c^{cre} *rufy3*$^{fl/fl}$ animal after intraperitoneal LPS injection (1,5ng/g). Scale bar is 2mm and 100 µm in box **c)** IHC staining in histological sections reveals B and T cells clustering in the lungs of CD11c^{cre} *rufy3*$^{fl/fl}$ animal after intraperitoneal LPS injection (1,5ng/g). Scale bar is 100 µm.

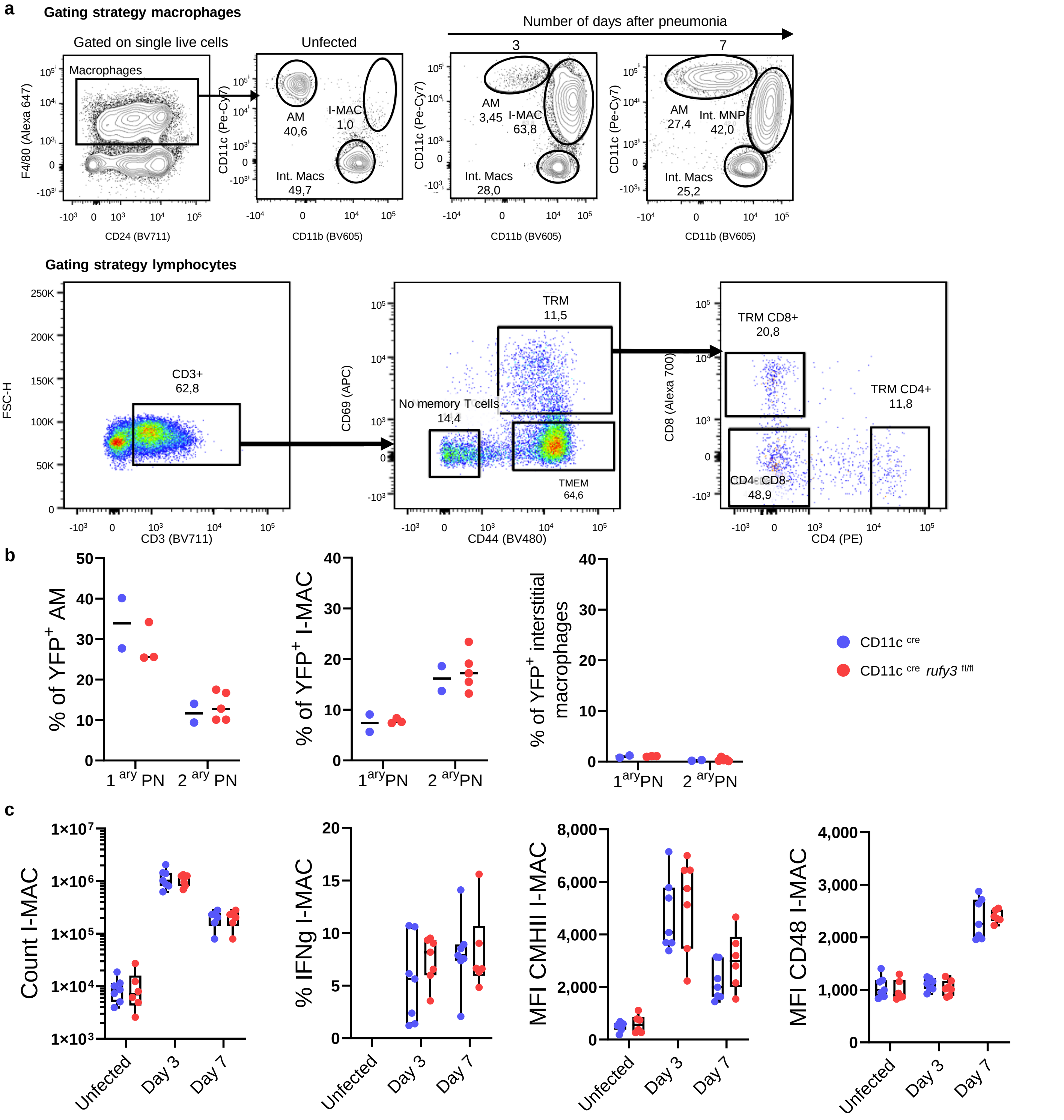

Supplementary Figure 7 Rufy3 deletion in the CD11C+ cell compartment is pro-inflammatory.

a) Gating strategy for flow analysis on lung during pneumonia. **b)** Phagocytic capacity of lung macrophages subsets during YFP+ *E. coli* infection during primary pneumonia (1^{ary} PN) and secondary pneumonia (2^{ary} PN) in CD11c^{cre} *rufy3 $^{fl/fl}$* mice. Data represent one dot for one mouse from two different experiments. **c)** Flow cytometry monitoring of lymphoid population during pneumonia in CD11c^{cre} *rufy3 $^{fl/fl}$* mice. N=7 for CD11c^{cre} mice and n=6 for CD11c^{cre} *rufy3 $^{fl/fl}$* mice. The boxplot data represent medians, interquartile ranges and spikes to upper and lower adjacent values.

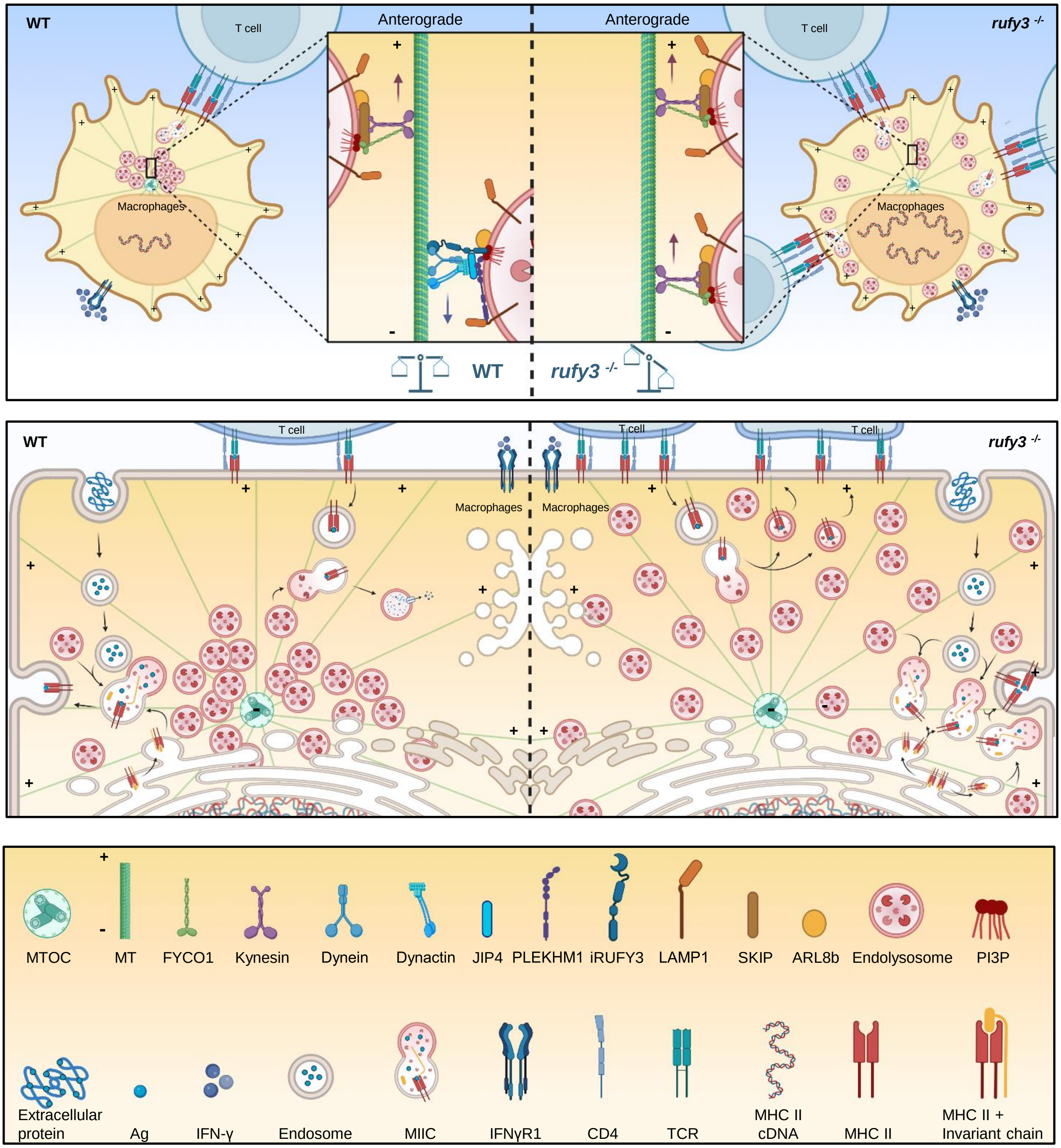

Supplementary Figure 8 Proposed model on the action of iRufy3 in endolysosomes dynamic.

Graphical model showing iRUFY3 function as an effector of ARL8b and new modulator of EL pericentriolar positioning. In the absence of iRUFY3, Dynein/Jip4-mediated ELs retrograde transport along microtubules (MT) is altered in activated macrophages. Abnormal peripheral EL positioning impacts globally endocytic functions with multiple consequences on macrophage biology, including resistance to *Salmonella* infection or inhibition of cell migration. Surprizingly, macrophages lacking iRUFY3 are more sensitive to IFN-γ stimulation and display reinforced MHC-II expression, invariant chain processing and proteolytic capacity, which translate into higher surface MHC II levels and strongly augmented antigen presentation to T cells. Created with Biorender©.

Source Data file Supplementary information table

Sup Fig 1c

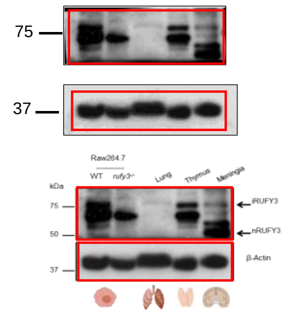

Sup Fig 1g

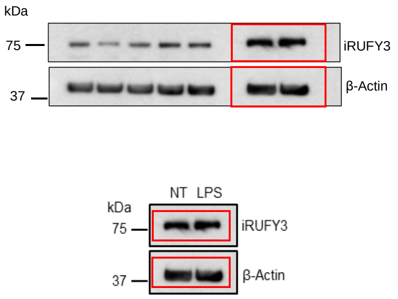

Sup Fig 5b

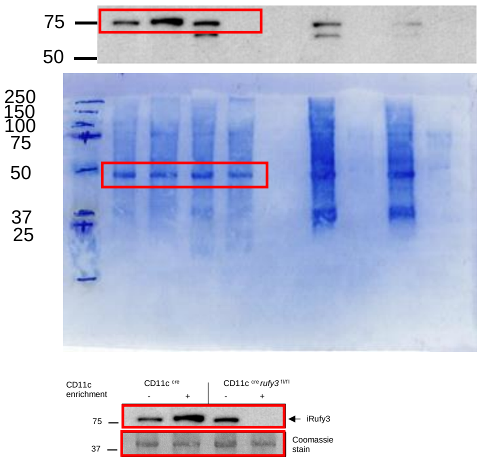

Sup Fig 5c

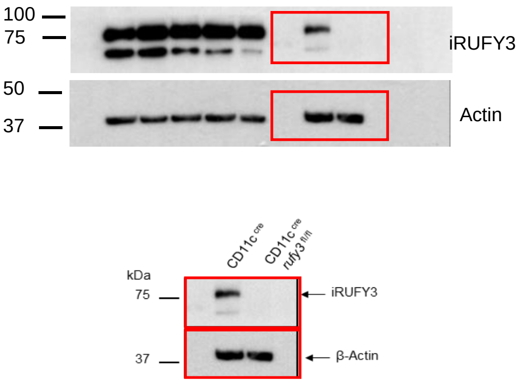

Sup Fig 5d

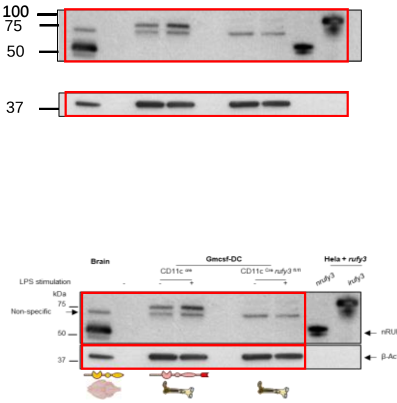

Sup Fig 5e

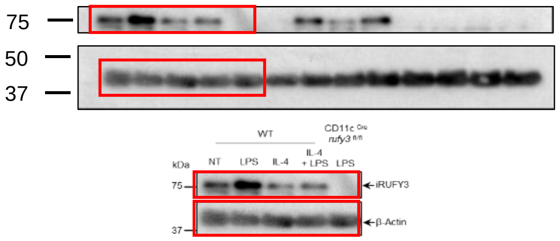

Supplementary Table 1: Primers list

Name	Forward Primer (5’ => 3’)	Reverse Primer (5’ => 3’)
Mouse *rufy3* long variant	GCTCTCTGAAAAGCCACAGG	CTTGACGGAAGAGGCAGTTC
Mouse *rufy3* short variant	TATGCCAACCCCCACCAC	TCTGTCAAATCCTCCCAAGAG
Mouse *fascin-1*	TTCCCTCTGTCCCCTTCTTT	GACGGGGTCTTCCAGTTACA
Mouse *fyco1*	TTCCTTTTCCCTCTCAAGCA	GCCACATGGATCGTGAATTA
Mouse *rufy1*	CTCTGTCTGAAGGACGCACA	TCGTCTTTTCCAAGCCATCT
Mouse *rufy2*	TCCACTCATTCCACCTTGCC	GGATACGGGTCTGTTGGGAG
Mouse *rufy4*	GCATAGGCTGCAACAAGGTC	CATCAGGGTTGGGAAGAGAA
Mouse *H-2 Iadα*	GTCAACCGTGACCATTCCTT	GGCACACACCACAGTTTCTG
Mouse *H-2 Iadβ*	ACCCAGCCAAGATCAAAGTG	ATCTCCAGCATGACCAGGAC
Mouse *CIITA*	ACACCTGGACCTGGACTCAC	GCTCTTGGCTCCTTTGTCAC
Mouse *IFNγR1*	TATTGTCGCTTCTGGCTCCT	AGACTTACGGCTGGCTTTGA
Rufy3 Screening primers	ATGGCCAGGAACAATACACC	ATGCCAAGCCCATTAACACT
Rufy3 sgRNA 1	caccgTCGTTAGCCATGAGATAATT	
Rufy3 sgRNA 2	aaacAATTATCTCATGGCTAACGAc	
Rufy3 sgRNA 3	caccgCACCTTTCAAGCCGTGTTTC	
Rufy3 sgRNA 4	aaacGAAACACGGCTTGAAAGGTGc	

Supplementary Table 2: Antibody panels for splenocytes immunophenotying.

Lymphoid panel (18 populations)									
**NK CD11b+Ly6C-**	CD161+	Ly6G-	CD317-	CD5-	CD11b+	Ly6C-			
**NK CD11b+Ly6C+**	CD161+	Ly6G-	CD317-	CD5-	CD11b+	Ly6C+			
**NK CD11b-Ly6C-**	CD161+	Ly6G-	CD317-	CD5-	CD11b-	Ly6C-			
**NK CD11b-Ly6C+**	CD161+	Ly6G-	CD317-	CD5-	CD11b-	Ly6C+			
**NKT**	CD161+	Ly6G-	CD317-	CD5+					
**B1 B cells**	CD161-	Ly6G-	CD317-	CD19+	MHCII+	CD5+			
**B2 B cells**	CD161-	Ly6G-	CD317-	CD19+	MHCII+	CD5-			
**B2 IgDlo**	CD161-	Ly6G-	CD317-	CD19+	MHCII+	CD5-	IgDlo		
**B2 IgD+**	CD161-	Ly6G-	CD317-	CD19+	MHCII+	CD5-	IgDhi		
**EM gd-T**	CD161-	Ly6G-	CD317-	CD5+	CD3+	TCRd+	CD44+	CD62L-	
**Naive gd-T**	CD161-	Ly6G-	CD317-	CD5+	CD3+	TCRd+	CD44-	CD62L+	
**CM gd-T**	CD161-	Ly6G-	CD317-	CD5+	CD3+	TCRd+	CD44+	CD62L+	
**EM CD4**	CD161-	Ly6G-	CD317-	CD5+	CD3+	TCRd-	CD4+	CD44+	CD62L-
**CM/Naive CD4**	CD161-	Ly6G-	CD317-	CD5+	CD3+	TCRd-	CD4+	CD44-	CD62L+
**Naive CD8**	CD161-	Ly6G-	CD317-	CD5+	CD3+	TCRd-	CD8+	CD44+	CD62L-
**CM CD8**	CD161-	Ly6G-	CD317-	CD5+	CD3+	TCRd-	CD8+	CD44-	CD62L+
**EM CD8**	CD161-	Ly6G-	CD317-	CD5+	CD3+	TCRd-	CD8+	CD44+	CD62L+
**Shedding**	CD161-	Ly6G-	CD317-	CD5+	CD3+	TCRd-	CD8+	CD44-	CD62L-

Myeloid panel (13 populations)														
**Neutrophils**	CD5-	CD11b+	Ly6G+											
**Eosinophils**	CD5-	Ly6G-	SigF+	SSChi										
**Monocytes**	CD5-	Ly6G-	SigF-	SSClo	CD11b+	Ly6C+								
**Macrophages**	CD5-	Ly6G-	SigF-	SSClo	CD11b+	Ly6C-	MHCII+/-	F4/80+	CD64+	CD16/32+				
**pDC**	CD5-	Ly6G-	SigF-	SSClo	CD11b+	Ly6C-	MHCII-	F4/80-	CD317+					
**cDC**	CD5-	Ly6G-	SigF-	SSClo	CD11b+	Ly6C-	MHCII+	F4/80-	CD317-	CD11c+				
**im cDC**	CD5-	Ly6G-	SigF-	SSClo	CD11b+	Ly6C-	MHCII+	F4/80-	CD317-	CD11c+	CCR2-	CD117+		
**Xcr1 Type im cDC**	CD5-	Ly6G-	SigF-	SSClo	CD11b+	Ly6C-	MHCII+	F4/80-	CD317-	CD11c+	CCR2-	CD117+	Xcr1+	CD11b-
**CD11b Type im cDC**	CD5-	Ly6G-	SigF-	SSClo	CD11b+	Ly6C-	MHCII+	F4/80-	CD317-	CD11c+	CCR2-	CD117+	Xcr1-	CD11b+
**mat cDC**	CD5-	Ly6G-	SigF-	SSClo	CD11b+	Ly6C-	MHCII+	F4/80-	CD317-	CD11c+	CCR2-	CD117-		
**Xcr1 Type mat cDC**	CD5-	Ly6G-	SigF-	SSClo	CD11b+	Ly6C-	MHCII+	F4/80-	CD317-	CD11c+	CCR2-	CD117-	Xcr1+	CD11b-
**CD11b+ Type mat cDC**	CD5-	Ly6G-	SigF-	SSClo	CD11b+	Ly6C-	MHCII+	F4/80-	CD317-	CD11c+	CCR2-	CD117-	Xcr1-	CD11b+
**NK**	CD5-	Ly6G-	CD161+	CD317-										
