# Supplementary material for: RUFY3 regulates endolysosomes perinuclear positioning, antigen presentation and migration in activated phagocytes
Source: Nat Commun. 2023 Jul 18;14:4290. doi: 10.1038/s41467-023-40062-x (PMC10354229; doi:10.1038/s41467-023-40062-x)
Supplement: Supplementary file 3 — Description of Additional Supplementary Files [file 41467_2023_40062_MOESM3_ESM.pdf]

Description of Additional Supplementary Files

File Name: Supplementary Data 1
Description: Tables of reagents and antibodies
