# Supplementary material for: RUFY3 regulates endolysosomes perinuclear positioning, antigen presentation and migration in activated phagocytes
Source: Nat Commun. 2023 Jul 18;14:4290. doi: 10.1038/s41467-023-40062-x (PMC10354229; doi:10.1038/s41467-023-40062-x)
Supplement: Supplementary file 5 — Reporting Summary [file 41467_2023_40062_MOESM5_ESM.pdf]

nature portfolio

Corresponding author(s): DBPR NCOMMS-22-40689-T

Last updated by author(s): Jun 19, 2023

Reporting Summary

Nature Portfolio wishes to improve the reproducibility of the work that we publish. This form provides structure for consistency and transparency in reporting. For further information on Nature Portfolio policies, see our Editorial Policies and the Editorial Policy Checklist.

Statistics

For all statistical analyses, confirm that the following items are present in the figure legend, table legend, main text, or Methods section.

n/a	Confirmed	
☐	☒	The exact sample size (*n*) for each experimental group/condition, given as a discrete number and unit of measurement
☐	☒	A statement on whether measurements were taken from distinct samples or whether the same sample was measured repeatedly
☐	☒	The statistical test(s) used AND whether they are one- or two-sided<br>*Only common tests should be described solely by name; describe more complex techniques in the Methods section.*
☒	☐	A description of all covariates tested
☒	☐	A description of any assumptions or corrections, such as tests of normality and adjustment for multiple comparisons
☐	☒	A full description of the statistical parameters including central tendency (e.g. means) or other basic estimates (e.g. regression coefficient) AND variation (e.g. standard deviation) or associated estimates of uncertainty (e.g. confidence intervals)
☐	☒	For null hypothesis testing, the test statistic (e.g. *F*, *t*, *r*) with confidence intervals, effect sizes, degrees of freedom and *P* value noted<br>*Give P values as exact values whenever suitable.*
☒	☐	For Bayesian analysis, information on the choice of priors and Markov chain Monte Carlo settings
☒	☐	For hierarchical and complex designs, identification of the appropriate level for tests and full reporting of outcomes
☐	☒	Estimates of effect sizes (e.g. Cohen's *d*, Pearson's *r*), indicating how they were calculated

Our web collection on statistics for biologists contains articles on many of the points above.

Software and code

Policy information about availability of computer code

Data collection	Microscopy images were acquired using Zein Black software v3.9 with Airyscan processing. For images analysis, Zen blue 3.5 was used (Carl Zeiss AG).<br>qPCR was performed ont Fast 7500 Fast real time PCR (Applied biossytems) with 7500 software v2.3.<br>For flow cytometry data collection, we used BD Canto II / LSRII UV/ LSR II symphony with BD diva software (v9.3) and exported the data in fcs3.0.

Data analysis

Data were plotted using Graph Pad Prism (Version 9.3.1, La Jolla, CA, United State). All statistical test and analysis were performed on Graph Pad Prism and were describe in the figures legends.
For confocal microscopy, he open source software mage J (V1.53u) were used with JACoP plugin to quantify colocalisation coefficient. For obtained clusturisation index and 360°ASOD data, a new plugin for Image J was created and available online (https://github.com/Imagimm-CIML/360-Angular-Scanning-monitoring-Organelle-Distribution)
For Three dimensional reconstitution images, Imaris software was used (v 9.9) from images acquired by airyscan.
For analysis on cytometry data, FLowJo software (v10.0) was used and numerical data exported to Excel sheet were further analyzing by using Graph pas Prism to generate graphical representation and for statistical analysis.
Sequences Alignments were performed with Sea View analysis software Version 5.0.5, https://evomics.org/resources/software/bioinformatics-software/seaview/
To obtain the Clustering index, a centroid based method was used with dedicated macro on ImageJ (https://github.com/Imagimm-CIML/Determining-cell-polarisation, Macro_polarisation_polar_dispersion.ijm).
360-ASOD (360° Angular Scanning monitoring Organelle Distribution) has been filed on Github (https://github.com/Imagimm-CIML/360-Angular-Scanning-monitoring-Organelle-Distribution

For manuscripts utilizing custom algorithms or software that are central to the research but not yet described in published literature, software must be made available to editors and reviewers. We strongly encourage code deposition in a community repository (e.g. GitHub). See the Nature Portfolio guidelines for submitting code & software for further information.

Data

Policy information about availability of data

All manuscripts must include a data availability statement. This statement should provide the following information, where applicable:

- Accession codes, unique identifiers, or web links for publicly available datasets
- A description of any restrictions on data availability
- For clinical datasets or third party data, please ensure that the statement adheres to our policy

All relevant data supporting this study'findings are presented in the manuscript and supplementary information. Raw data, uncropped western blot are available in the Source Data file which is provided with the manuscript.
Due to the large number of images and their consequent size, microscopy images are available on request from the author, microscopy data that support our finding are available on reasonable request from the corresponding author.
Gene description and sequences are available from NCBI databases: https://www.ncbi.nlm.nih.gov/gene?Db=gene&Cmd=DetailsSearch&Term=52822
Gene expression profiles from immune cells are established with Immunological Genome project: https://www.immgen.org

Human research participants

Policy information about studies involving human research participants and Sex and Gender in Research.

Reporting on sex and gender: No human participants was involved in this research

Population characteristics: No human participants was involved in this research

Recruitment: No human participants was involved in this research

Ethics oversight: No human participants was involved in this research

Note that full information on the approval of the study protocol must also be provided in the manuscript.

Field-specific reporting

Please select the one below that is the best fit for your research. If you are not sure, read the appropriate sections before making your selection.

☒ Life sciences ☐ Behavioural & social sciences ☐ Ecological, evolutionary & environmental sciences

For a reference copy of the document with all sections, see nature.com/documents/nr-reporting-summary-flat.pdf

Life sciences study design

All studies must disclose on these points even when the disclosure is negative.

Sample size

All sample sizes are listed in detail in the figure legends of the relevant figures. Samples sizes were determined bases on the estimates from preliminary experiments and similar studies (Kumar et al, Nature communication (2022) and Roquilly et al, Nature Immunology (2020)) so that reasonable statistical analysis could be conducted. A minimum of three biological replicates were taken for each experimental set, unless otherwise mentioned.

Data exclusions

For velocity assay, outlier cells with exacerbated speed were excluded.
For microscopy, Binucleated cells and non--uniform shaped cells were excluded from analysis.

Replication	Experiments were conducted multiple times and were reproducible, as indicated in the legends to each figures.
Randomization	Randomization was not performed as all the cells were imaged and analyzed in the same way.<br>For all experiments on mice, mice were ear tagged and randomly assigned into experimental groups, and littemate controls were used where appropriate.
Blinding	On cell lines, blinding was not performed as cell were prepared and analyzed by the same investigator<br>For the mice, blinding was performed with only the operator knowing that the mice (ear-marked) were referenced by number. The investigator continued the experiment and analysis without knowing which mice referred to which group.

Reporting for specific materials, systems and methods

We require information from authors about some types of materials, experimental systems and methods used in many studies. Here, indicate whether each material, system or method listed is relevant to your study. If you are not sure if a list item applies to your research, read the appropriate section before selecting a response.

Materials & experimental systems

n/a	Involved in the study
☐	☒ Antibodies
☐	☒ Eukaryotic cell lines
☒	☐ Palaeontology and archaeology
☐	☒ Animals and other organisms
☒	☐ Clinical data
☒	☐ Dual use research of concern

Methods

n/a	Involved in the study
☒	☐ ChIP-seq
☐	☒ Flow cytometry
☒	☐ MRI-based neuroimaging

Antibodies

Antibodies used

All antibodies used were used for the following antigens (clones) at the stated concentrations and from the stated vendor (lot number). Table is described in supplemental table 1 :

Confocal microscopy and western blot :

Anti-goat IgG Alexa Fluor 555, donkey, 1:1 000 Invitrogen Cat#A-11057
Anti-mouse ARL8B, Rabbit IgG, 1:100 Proteintech Cat#13049-1-AP
Anti-mouse B220 (Clone: RA3-6B2) pure, rat, 1:100 Biolegend Cat#103251
Anti-mouse β-actin (Clone: AC15), mouse IgG1, 1:20 000 Merck Cat#A5441-100UL
Anti-mouse CD3 (Clone 17A2) pure, rat, 1:100 Biolegend Cat#100202
Anti-mouse EEA1, goat, 1:100, MyBiosource, Cat#423213
Anti-mouse IgG1 Alexa Fluor 488, goat, 1:1 000 Invitrogen Cat#A-21121
Anti-mouse IgG1 Alexa Fluor A568, goat , 1:1 000 Invitrogen Cat#A-21124
Anti-mouse IgG2a Alexa Fluor 488, goat, 1:1 000 Invitrogen Cat#A21131
Anti-mouse IgG2a Alexa Fluor 568, goat, 1:1 000 Invitrogen Cat#A21134
Anti-mouse LAMP1 Alexa Fluor 647 (Clone: 1D4B), rat IgG2b, 1:100 Biolegend Cat#121610
Anti-mouse LAMP2 (Clone: GL2A7), rat IgG2b, 1:100 Invitrogen Cat#MA1-165
Anti-mouse LC3B (Clone: 2G6), mouse IgG1, 1:20 NanoTools Cat#0260-100
Anti-mouse MHC-II (Clone M5/114.15.2), rat, 1:100 eBioscience Cat#14-5321-52
Anti-mouse Myc (Clone: 9B11), mouse IgG2a, 1:1 000 Cell singaling Cat#2276
Anti-mouse p62, guinea pig IgG, 1:200 Progen Cat#GP62-C
Anti-mouse Rab11A (Clone: 3H18L5), rabbit IgG, 1:100 Invitrogen Cat#700184
Anti-mouse RIPX/RUFY3, rabbit IgG, 1:1000 Novus Biological NBP1-89614
Anti-mouse Syntaxin6, rabbit IgG, 1:100 Proteinteich Cat#1034-1-AP
Anti-mouse IgG HRP, goat, 1:5000 Invitrogen Cat#31430
Anti-mouse p62/SQSTM1 (Clone: D-3), mouse IgG1, 1:100 Santa Cruz Biotech Cat#sc-28359
Anti-rabbit IgG Alexa Fluor 568, goat, 1:1 000 Invitrogen Cat#A-21428
Anti-rabbit IgG Alexa Fluor 555, goat, 1:1 000 Invitrogen Cat#A-11011
Anti-rabbit IgG HRP, goat, 1:10 000 Invitrogen Cat#31460
Anti-rat IgG Alexa Fluor 647, goat, 1:1 000 Invitrogen Cat#A-21247
Anti-rat IgG Alexa Fluor 488, goat, 1:1 000 Jackson Immunoresearch Cat#712-545-153
Anti-rat IgG Alexa Fluor 555, goat, 1:1 000 Invitrogen Cat#A-21434

Flox cytometry :

Anti-mouse CD3 (Clone 17A2) BV711,rat, 1:200 BD Bioscience Cat#740739
Anti-mouse CD3 (Clone: 145-2C11) BV421, rat, 1:100 Biolegend Cat#BLE100335
Anti-mouse CD4 (Clone GK1.5) PE, rat, 1:200 Biolegend Cat#100408
Anti-mouse CD8 (Clone 3B5) Alexa Fluor 700, mouse IgG2a, 1:200 Invitrogen Cat#MHCD0829
Anti-mouse CD11b (Clone M1/70) BV605, rat, 1:200 BD Bioscience Cat#8190980
Anti-mouse CD11c (Clone N418) PE-Cy7, rat, 1:400 Biolegend Cat#B222652

Anti-mouse CD16/CD32 Mouse BD Fc Block™ (Clone: 2.4G2), rat, 1:200 BD Pharmigen Cat#553142
Anti-mouse CD24 (Clone M1/69) BV711, rat, 1:200 BD Bioscience Cat 9043567
Anti-mouse CD44 (Clone IM7) BV480, rat, 1:200 BD Bioscience Cat#566200
Anti-mouse CD69 (Clone H1.2F3) APC, Hamster 1:200 Biolegend Cat#104514
Anti-mouse CD86 (Clone GL-1) APC, rat, 1:300 Biolegend Cat#105012
Anti-mouse CD103 (Clone 2E7) BV605, hamster 1:100 BD Bioscience Cat#748257
Anti-mouse CD172a (SIRP-α, Clone P84) PE, rat, 1:100 BD Bioscience Cat#5191597
Anti-mouse CXCR6 (Clone SA051D1) PE-Dazzle, rat 1:100 Biolegend Cat#151117
Anti-mouse F4/80 (Clone BM8) BV711, rat, 1:200 Biolegend Cat#123147
Anti-mouse IFN-γ (Clone XMG1.2) PE, rat, 1:200 BD Bioscience Cat#554412
Anti-mouse Ly-6C (Clone: HK1.4) BV711, rat, 1:200 Biolegend Cat#128037
Anti-mouse Ly-6C (Clone AL-21) FITC, rat, 1:200 BD Bioscience Cat#553104
Anti-mouse Ly6-G (Clone: 1A8) PE-Cy7, rat, 1:200 Biolegend Cat#127618
Anti-mouse MHC I (clone SF1-1.1) PE, mouse IgG2a, 1:200 Biolegend Cat#116608
Anti-mouse MHC-II (Clone M5/114.15.2) BV480, rat, 1:200 BD Bioscience Cat#566088
Anti-mouse MHC-II (Clone: M5/114.15.2) Alexa Fluor 700, rat, 1:300 eBioscience Cat#56-5321-82
Anti-mouse MHC-II (Clone M5/114.15.2) BUV805, rat, 1:500 BD Bioscience Cat#748844
Anti-mouse NK1.1 (Clone PK136) BV421, mouse IgG2a 1:200 Biolegend Cat#108741
Anti-mouse NK1.1 (Clone PK136) BV510, mouse IgG2a, 1:300 Biolegend Cat#108373
Anti-mouse TCR γδ (Clone 11F2) PE-Cy7, 1:200 BD Bioscience Cat#655410

Validation

The following monoclonal antibodies were used (name, clone, species, validation dilution and cat# supplier) with RRID associated number; Where no RRID is provided here staining agents were validated and titrated inhouse and/or according to manufacturer's specifications. All the other antibodies lacking RRID were clones that have been extensively used in the literature. We also titrated all antibodies prior to experiments.

Confocal microscopy and western blot :

Anti-mouse ARL8B, Rabbit IgG, 1:100 Proteintech Cat#13049-1-AP RRID:AB_2059000
Anti-mouse B220 (Clone: RA3-6B2) pure, rat, 1:100 Biolegend Cat#103251 RRID:AB_2562905
Anti-mouse β-actin (Clone: AC15), mouse IgG1, 1:20 000 Merck Cat#A5441-100UL RRID:AB_476744
Anti-mouse CD3 (Clone 17A2) pure, rat, 1:100 Biolegend Cat#100202 RRID:AB_312659
Anti-mouse EEA1, goat, 1:100, MyBiosource, Cat#423213
Anti-mouse LAMP1 Alexa Fluor 647 (Clone: 1D4B), rat IgG2b, 1:100 Biolegend Cat#121610 RRID:AB_571991
Anti-mouse LAMP2 (Clone: GL2A7), rat IgG2b, 1:100 Invitrogen Cat#MA1-165 RRID:AB_2609332
Anti-mouse LC3B (Clone: 2G6), mouse IgG1, 1:20 NanoTools Cat#0260-100
Anti-mouse MHC-II (Clone M5/114.15.2), rat, 1:100 eBioscience Cat#14-5321-52 RRID:AB_1123540
Anti-mouse Myc (Clone: 9B11), mouse IgG2a, 1:1 000 Cell singaling Cat#2276 RRID:AB_331783
Anti-mouse p62, guinea pig IgG, 1:200 Progen Cat#GP62-C RRID:AB_1542690
Anti-mouse Rab11A (Clone: 3H18L5), rabbit IgG, 1:100 Invitrogen Cat#700184 RRID:AB_2532295
Anti-mouse RIPX/RUFY3, rabbit IgG, 1:1000 Novus Biological NBP1-89614 RRID:AB_11022810
Anti-mouse Syntaxin6, rabbit IgG, 1:100 Proteinteich Cat#1034-1-AP
Anti-mouse p62/SQSTM1 (Clone: D-3), mouse IgG1, 1:100 Santa Cruz Biotech Cat#sc-28359 RRID:AB_628279

Flow cytometry :

Anti-mouse CD3 (Clone 17A2) BV711,rat, 1:200 BD Bioscience Cat#740739 RRID:AB_2740409
Anti-mouse CD3 (Clone: 145-2C11) BV421, rat, 1:100 Biolegend Cat#BLE100335 RRID:AB_627016
Anti-mouse CD4 (Clone GK1.5) PE, rat, 1:200 Biolegend Cat#100408 RRID:AB_1117291
Anti-mouse CD8 (Clone 3B5) Alexa Fluor 700, mouse IgG2a, 1:200 Invitrogen Cat#MHCD0829 RRID:AB_10372957
Anti-mouse CD11b (Clone M1/70) BV605, rat, 1:200 BD Bioscience Cat#8190980 RRID:AB_2870249
Anti-mouse CD11c (Clone N418) PE-Cy7, rat, 1:400 Biolegend Cat#B222652 RRID:AB_2534619
Anti-mouse CD16/CD32 Mouse BD Fc Block™ (Clone: 2.4G2), rat, 1:200 BD Pharmigen Cat#553142 RRID:AB_394657
Anti-mouse CD24 (Clone M1/69) BV711, rat, 1:200 BD Bioscience Cat#9043567 RRID:AB_626988
Anti-mouse CD44 (Clone IM7) BV480, rat, 1:200 BD Bioscience Cat#566200 RRID:AB_2739591
Anti-mouse CD69 (Clone H1.2F3) APC, Hamster 1:200 Biolegend Cat#104514 RRID:AB_492843
Anti-mouse CD86 (Clone GL-1) APC, rat, 1:300 Biolegend Cat#105012 RRID:AB_493342
Anti-mouse CD103 (Clone 2E7) BV605, hamster 1:100 BD Bioscience Cat#748257 RRID:AB_2872686
Anti-mouse CD172a (SIRP-α, Clone P84) PE, rat, 1:100 BD Bioscience Cat#5191597 RRID:AB_11149864
Anti-mouse CXCR6 (Clone SA051D1) PE-Dazzle, rat 1:100 Biolegend Cat#151117 RRID:AB_2721700
Anti-mouse F4/80 (Clone BM8) BV711, rat, 1:200 Biolegend Cat#123147 RRID:AB_2564588
Anti-mouse IFN-γ (Clone XMG1.2) PE, rat, 1:200 BD Bioscience Cat#554412 RRID:AB_395376
Anti-mouse Ly-6C (Clone: HK1.4) BV711, rat, 1:200 Biolegend Cat#128037 RRID:AB_2562630
Anti-mouse Ly-6C (Clone AL-21) FITC, rat, 1:200 BD Bioscience Cat#553104 RRID:AB_394628
Anti-mouse Ly6-G (Clone: 1A8) PE-Cy7, rat, 1:200 Biolegend Cat#127618 RRID:AB_1877261
Anti-mouse MHC I (clone SF1-1.1) PE, mouse IgG2a, 1:200 Biolegend Cat#116608 RRID:AB_313743
Anti-mouse MHC-II (Clone M5/114.15.2) BV480, rat, 1:200 BD Bioscience Cat#566088 RRID:AB_2869739
Anti-mouse MHC-II (Clone: M5/114.15.2) Alexa Fluor 700, rat, 1:300 eBioscience Cat#56-5321-82 RRID:AB_494009
Anti-mouse MHC-II (Clone M5/114.15.2) BUV805, rat, 1:500 BD Bioscience Cat#748844 RRID:AB_2873247
Anti-mouse NK1.1 (Clone PK136) BV421, mouse IgG2a 1:200 Biolegend Cat#108741 RRID:AB_2562561
Anti-mouse NK1.1 (Clone PK136) BV510, mouse IgG2a, 1:300 Biolegend Cat#108373 RRID:AB_2562216
Anti-mouse TCR γδ (Clone 11F2) PE-Cy7, 1:200 BD Bioscience Cat#655410 RRID:AB_2870377

Eukaryotic cell lines

Policy information about cell lines and Sex and Gender in Research

Cell line source(s)	RAW 264.7 were obtained from America Type Culture Collection (ATCC) and expended following their instruction. Raw 264.7 rufy3 KO and complemented irufy or nrufy3 RAW cells were generated as indicated in the material & method section of the manuscrit).
Authentication	All cell lines used in this study were authenticated by ATCC and morphology was assessed by microscopy. Cells were cultured for no more than 15 passages.
Mycoplasma contamination	All cell lines used in this study were regularly screened for the absence of mycoplasma contamination by using the MycoAlert Mycoplasma Detection Kit (LT07-418, Lonza) and we confirm that all cell used in this study tested negative.
Commonly misidentified lines (See ICLAC register)	No misidentified cell lines were used in the study.

Animals and other research organisms

Policy information about studies involving animals; ARRIVE guidelines recommended for reporting animal research, and Sex and Gender in Research

Laboratory animals	C57BL/6 mice were purchased from Janvier labs and bred in-house. CD11c-Cre and CD11c-Cre-rufy3-flox/flox were bred in CIPHE animals house. Matched male or female mice, aged 6 to 12weeks old, were used.<br>All the mice were bred and maintained under specific-pathogen-free conditions at the Centre d'Immunophénomique (Ciphe) de Marseille and at the Centre d'Immunologie de Marseille Luminy (CIML). Mice were housed under a standard 12 h:12 h light:dark cycle with ad libitum access to food and water. Age-matched (6–12 weeks old, unless otherwise specified) and sex-matched littermate mice were used as controls.
Wild animals	Study did not involved wild animals
Reporting on sex	For all experiments using mice, males and females were used. Preliminary results prove that not gender effect were associated to the study. For each experiment, same number of male and female of matched aged, were used. All mice were randomized and unique ID number was attributed to a mouse, without taking account about the gender.
Field-collected samples	Study did not involved samples collected from the field
Ethics oversight	All experiments were approved by the "Comité d'Ethique PACA" and MESRI (approval number APAFIS#18981-2019020710111763)

Note that full information on the approval of the study protocol must also be provided in the manuscript.

Flow Cytometry

Plots

Confirm that:

☒ The axis labels state the marker and fluorochrome used (e.g. CD4-FITC).

☒ The axis scales are clearly visible. Include numbers along axes only for bottom left plot of group (a 'group' is an analysis of identical markers).

☒ All plots are contour plots with outliers or pseudocolor plots.

☒ A numerical value for number of cells or percentage (with statistics) is provided.

Methodology

Sample preparation	Following euthanasia of animals, spleens and lungs were removed, crushed with GentlaMACS Octo dissociator and digested at 37°C in RPMI 5% FCS containing 5mg/mL liberase and 150%g/mL DNase I for 30 min. The digestion was stopped by additing EDTA (Sigma) of a final concentration oh 5mM was passed through a 70µm cell strainer. After red blood cell lysis, cells were processed for flow cytometry.
Instrument	Sample were acquired using a 3 laser Canto II or 4 laser LSRII-UV or symphony (BD Bioscience). CD11c+ cells were sorted with AutoMACS pro Separator from Miltenyi
Software	Samples were acquired with BD FACSDiva v 8.0.1 software and analyzed with FlowJo software (v10, Tree star)
Cell population abundance	To study RAW264.7 cell lines, macrophages accounted for 90%of single living cells in culture after treatment.<br>Alveolar macrophages accounted for 10-15% of single lining cells in lung homogenates obtained after mechanistical and enzymatic tissue digestion.<br>After sorting on autoMACS pro separator, CD11c+ cells were sorted to a purity of 95-98% (post-sort analysis for purity determination)

Gating strategy

MHC-II surface expression and OVA-DQ in cell lines :
Doublets were excluded by plotting the height against the area for forward scatter and dead cells (fixable viability dye) were excluded, prior MHC-II and BODIPY intensity monitoring.

LPS intra-peritoneal injection :
First, doublets (by plotting the height against the area for forward scatter) and dead cells (fixable viability dye) were excluded. Then cells population were identifyied with specific markers. List of all specific markers and profile for each population was detailed in supplementary table 3 (Lymphoïd and Myeloïid panel).

Pneumonia experiments :
First, doublets (by plotting the height against the area for forward scatter) and dead cells (fixable viability dye) were excluded. Alveolar macrophages and Inflammatory macrophages were identified by a sequential gating strategy based on the expression of specific markers (F4/80, CD11c, CD11b). T cell sub-population were identified by a sequential gating strategy bases on the expression of specific markers (CD3, CD4, CD8 CD69, CD44) Supplementary figure 9 exemplifies the gating strategy.

☒ Tick this box to confirm that a figure exemplifying the gating strategy is provided in the Supplementary Information.
